# rTMS alleviates AD-induced cognitive impairment by inhibitng apoptosis in SAMP8 mouse

**DOI:** 10.18632/aging.203796

**Published:** 2021-12-29

**Authors:** Zheng Bao, Li Bao, Na Han, Yueyun Hou, Fumin Feng

**Affiliations:** 1 School of Public Health, North China University of Science and Technology, Tangshan 063210, Hebei Province, P.R. China; 2 Child Health Division, Tongzhou Maternal and Child Health Hospital of Beijing, Beijing 101101, P.R. China; 3 College of Life Science, North China University of Science and Technology, Tangshan 063210, Hebei, P.R. China; 4 Langfang Guangyang District People's Hospital, Langfang 065099, Hebei, P.R. China

**Keywords:** Alzheimer’s disease, cognitive impairment, rTMS, apoptosis, cAMP/PKA/CREB signaling pathway

## Abstract

This study sought to investigate whether repetitive transcranial magnetic stimulation (rTMS) could alleviate cognitive dysfunction in SAMP8 mice by reducing cell apoptosis and activating the cAMP/PKA/CREB signalling pathway. A total of 40 SAMP8 mice were randomly assigned to the SAMP8 group (n=20), and rTMS treatment group (rTMS+SAMP8, n=20); additionally, 20 homologous and normal aged SAMR1 mice were used as the control group(n=20). The Morris water maze and Y maze tests were applied to evaluate spatial learning and memory ability. Haematoxylin and eosin (HE) staining and terminal-deoxynucleotidyl transferase-mediated nick end labelling (TUNEL) were used to observe the changes in neurons in the cortex and hippocampus. Western blotting and RT-PCR were used to detect signalling related proteins. rTMS significantly improved spatial learning and memory deficits and morphological abnormalities in the hippocampus region of the hippocampus. In addition, rTMS reduced apoptosis of neurons caused by AD and the expression of pro-apoptotic proteins (Caspase-3 and Bax) and increased the expression of an antiapoptotic protein (Bcl-2). Furthermore, rTMS activated the cAMP/PKA/CREB signalling pathway. These results showed that rTMS could ameliorate cognitive deficits in AD mice by inhibiting apoptosis via activation the cAMP/PKA/CREB signalling pathway.

## INTRODUCTION

As the life expectancy of the Chinese population increases, the number of patients with Alzheimer's disease will increase [1, 2]. At present, in developed countries, with the rapid ageing of the population, AD, together with heart disease, cancer and cerebrovascular disease, have become major diseases threatening the health and life of the elderly [3]. The incidence of AD is on the rise, bringing a large burden to patients, their families and society [4]. China’s ageing population is developing rapidly. It is estimated that by 2050, elderly population of AD patients in this country will reach 20 million. China is about to become the region with the largest number of AD patients and the fastest growth rate in the world. Thus, AD may become a major disease threatening the health of the elderly population in China [2]. Therefore, the strengthening of AD research not only has great significance for China, but also will have a profound historical impact. AD is the most common form of dementia and is characterized by loss of memory, language and behaviour abilities [5]. It has been reported that AD accounts for up to 80% of all types of dementia [6]. AD is a manifestation of brain ageing, and the pathology is extremely complex [7]. The structural changes include brain weight and volume reduction, neuronal volume reduction, neuronal selective loss, and neuronal protrusion reduction [8, 9]. A large amount of evidence has shown that apoptosis is the ultimate fate of nerve cells in many neurodegenerative diseases, including AD, and its excessive apoptosis is one of the main causes of AD [10, 11]. At present, there is no effective method to combat AD, so the prevention and treatment of AD has become a focus of neuroscience research.

Repetitive transcranial magnetic stimulation (rTMS) is a non-invasive, electrodeless brain stimulation technique that uses a time-varying magnetic field to generate induced currents to directly stimulate cortical neurons and adjust the function of the latter [12]. rTMS can affect the excitability of the central nervous system by adjusting its frequency, intensity, stimulation interval and duration. For more than 20 years, rTMS has been widely used in various fields such as nerves, spirit, and psychology [13], and has made significant contributions to the understanding of perception, attention, consciousness, cortical function connections and plasticity. At present, the phenomenon of social ageing is very serious. Dementia and cognitive dysfunction are among the research hotspots of the 21st century. Future rTMS research should mainly focus on the mechanistic research and treatment effects of neuropsychiatric diseases, such as degenerative diseases of the nervous system [14, 15]. The common symptoms of learning and memory decline are treatment effects. This study explored rTMS to observe how high-frequency rTMS affects the cognitive function of AD, to provide a certain basis for the clinical prevention and treatment of AD.

## RESULTS

### Improvement in cognitive function of AD mice after rTMS treatment

**Figure 1:**
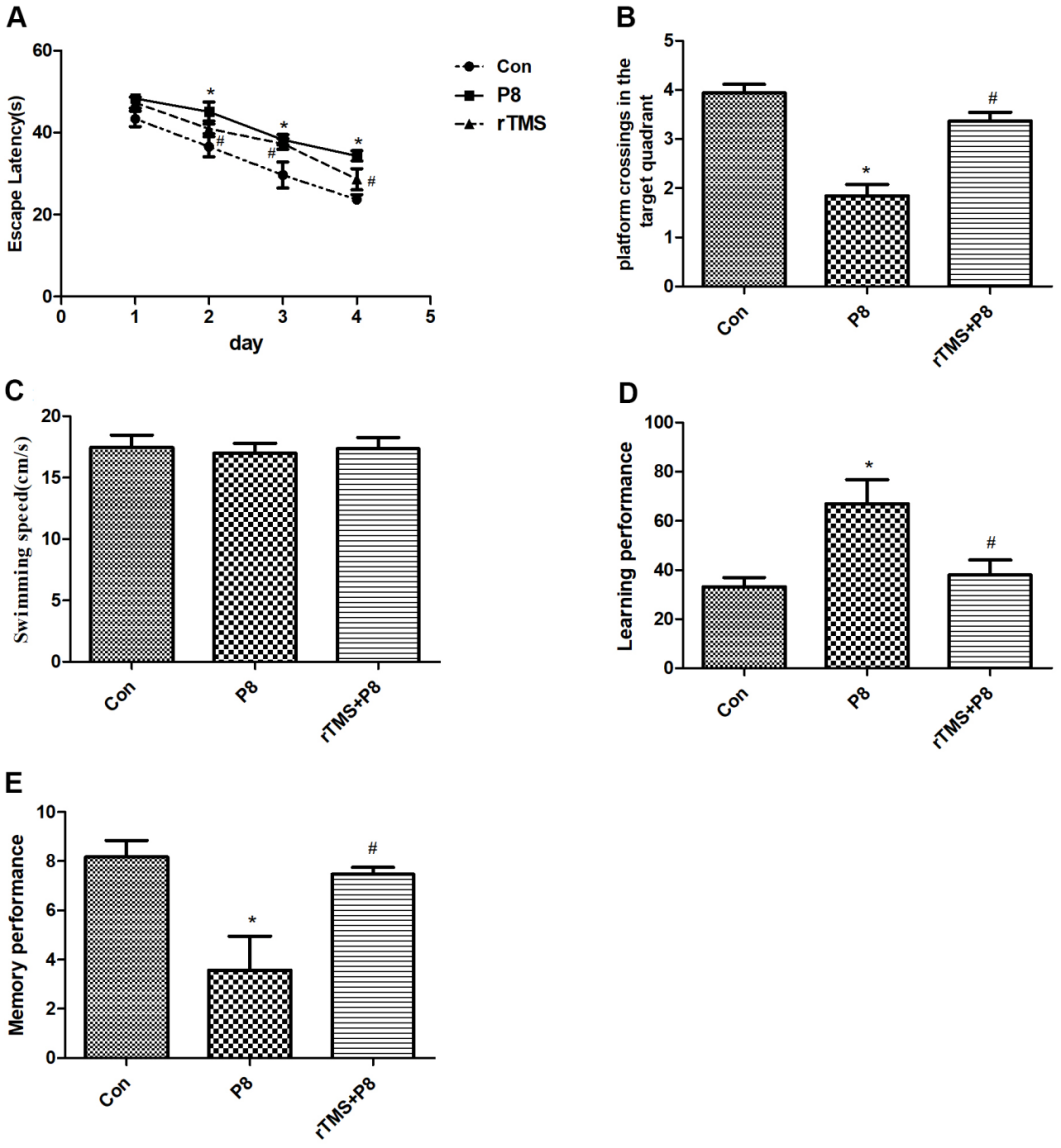
**Improvement in cognitive function of the mice after rTMS treatment.** (**A**) The latency time was compared between the three groups. (**B**) The dwell time in the target quadrant of mice in the three groups was compared. (**C**) The mean swimming speed of mice in three groups is shown. (**D**) The learning performance of mice in the three groups was compared. (**E**) The memory performance of mice in the three groups was compared. The data is demonstrated as the mean ± standard deviation (n =20). ^*^P < 0.05 vs. Con group; ^#^ *P* < 0.05 vs. P8 group.

To exclude the effects of rTMS on mice with cognitive deficits, MWM test was performed at 1-5 days after rTMS treatment in the three groups. In the orientation navigation experiment, we found significant differences in latency time between the Con group and P8 group, and the latency time of the P8 group was much longer than that of the Con group. However, the latency time was significantly decreased in the rTMS group compared with the P8 group (Figure 1A). In the spatial probe test, compared with the Con group, the platform crossings of the P8 group in the target quadrant were shortened, while rTMS prolonged the crossings of P8 mice in the platform quadrant (Figure 1B). However, no difference in swimming speed was observed between the three groups (Figure 1C). The Y-maze test was performed to assess the cognitive function of all animals. Compared with the Con group, the number of training required to reach the 9/10 standard in the P8 group was significantly increased, and the learning and memory performance was significantly decreased; the learning and memory scores of the rTMS group were significantly improved compared with those of the p8 group (Figure 1D, 1E). The results showed that rTMS improved cognitive disorders of in Alzheimer's disease which improved the performance of the mice.

### Reduction in the apoptosis of cortical and hippocampal neurons of P8 mice after rTMS treatment

**Figure 2:**
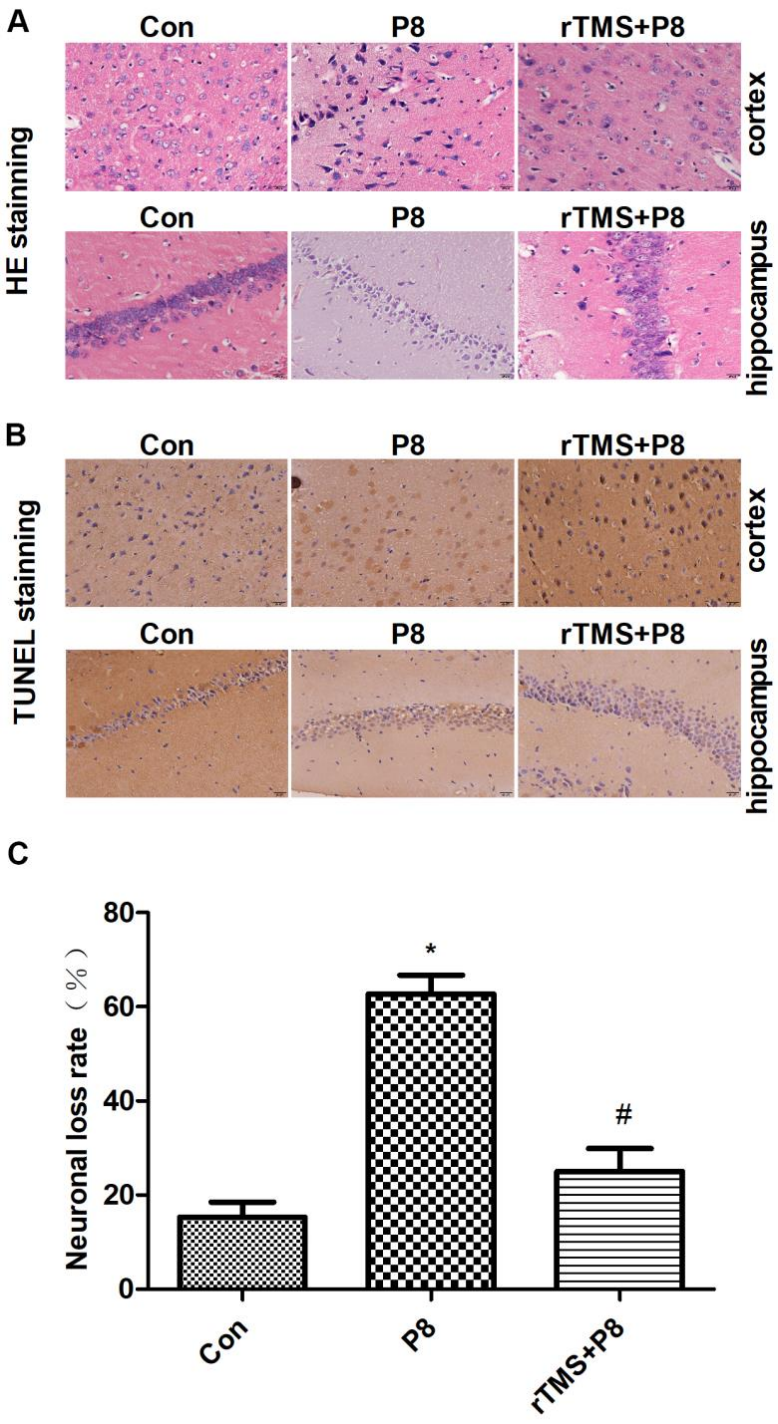
**Reduction in neuronal loss in the cortical and hippocampal neurons of P8 mice after rTMS treatment.** (**A**) The changes in the morphology of cortical and hippocampal neurons in mice were evaluated by HE staining (scale bar, 20μm). (**B**) TUNEL staining was performed for the mouse cortical and hippocampal regions (scale bar, 20μm, magnification, 10x eye piece x 40x objective). (**C**) Histogram analysis of TUNEL-positive cells. The data is demonstrated as the mean ± standard deviation (n =5). *P < 0.05 vs. Con group; # P < 0.05 vs. P8 group. TUNEL, the terminal deoxynucleotidyl transferase nick-labelling; HE, haematoxylin and eosin.

Morphological changes in cortical and hippocampal neurons were observed by HE staining. As shown in the HE staining results (Figure 2A), cortical and hippocampal neurons in the Con group were arranged in an orderly manner, chromatin was evenly distributed and nucleoli were clearly visible. In the P8 group, we observed a completely opposite pattern to the Con group. After rTMS treatment, the levels of neuron degeneration, necrosis, and loss were reduced compared with those in the P8 group. Then, TUNEL staining was used to observe the effect of rTMS on the apoptosis of cortical and hippocampal cells. As shown in Figure 2B, 2C, a small number of cortical and hippocampal neurons in the control group suffered apoptosis. In the P8 group, disordered neuronal cell arrangement was observed and the number of apoptotic cortical and hippocampal neurons increased. After rTMS treatment, the apoptotic neurons of P8 mice were significantly reduced. The results indicated that the improvement of learning and memory impairment by rTMS may be related to the reduction of neuron loss.

### Changes in the expression levels of apoptosis-related proteins and mRAN in the P8 mouse cortex and hippocampus after rTMS treatment

**Figure 3:**
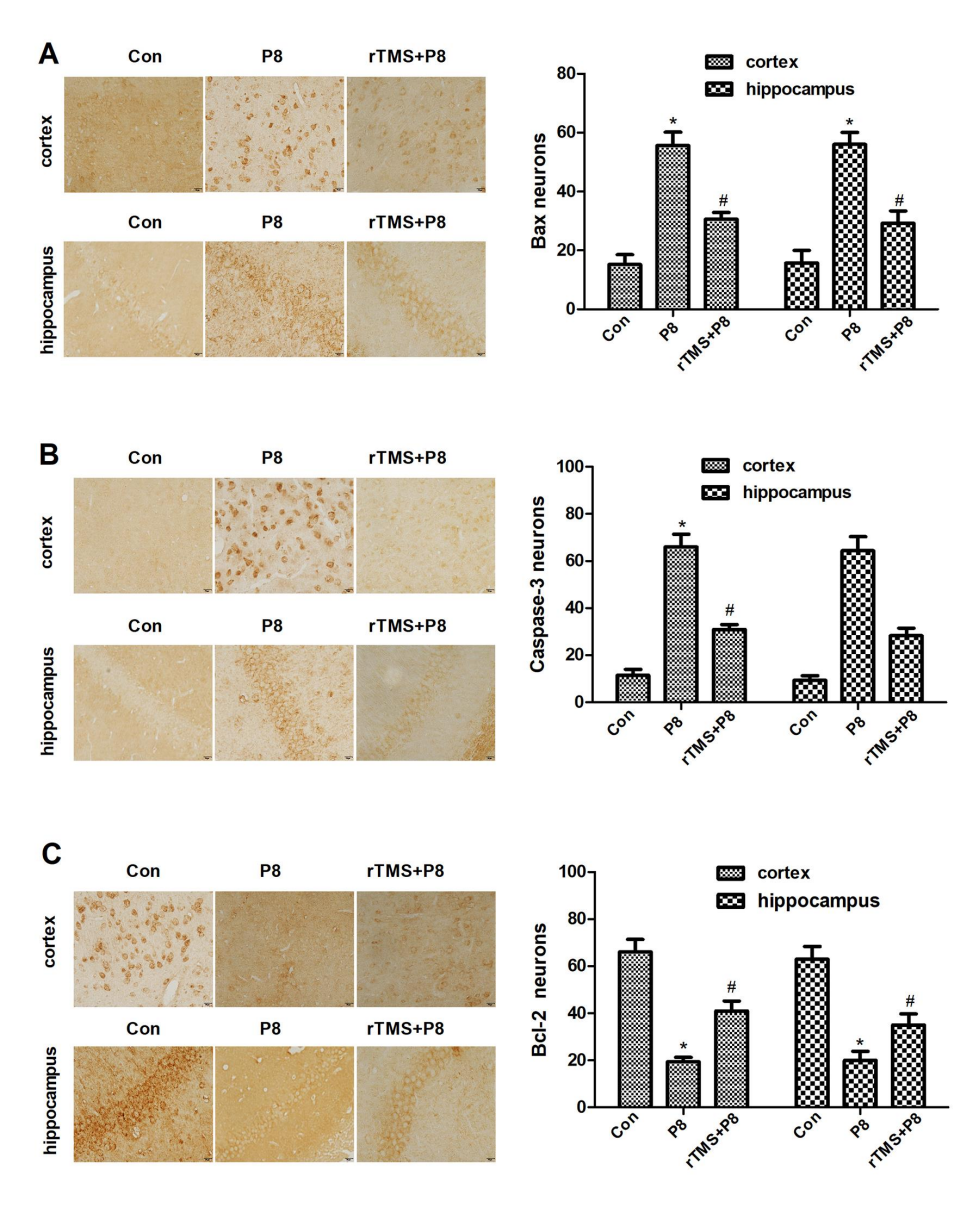
**Changes in the expression levels of apoptosis-related proteins in cortical and hippocampal regions in the three groups after rTMS treatment.** (**A**) Bax expression in the three groups was examined by immunohistochemistry staining and quantitative histogram analysis. (**B**) Caspase-3 expression in the three groups was examined by immunohistochemistry staining in three groups and quantitative histogram analysis. (**C**) Bcl-2 expression- in the three groups was examined by immunohistochemistry staining and quantitative histogram analysis. The data are presented as the mean ± standard deviation (n =5). *P < 0.05 vs. Con group; # P < 0.05 vs. P8 group.

**Figure 4:**
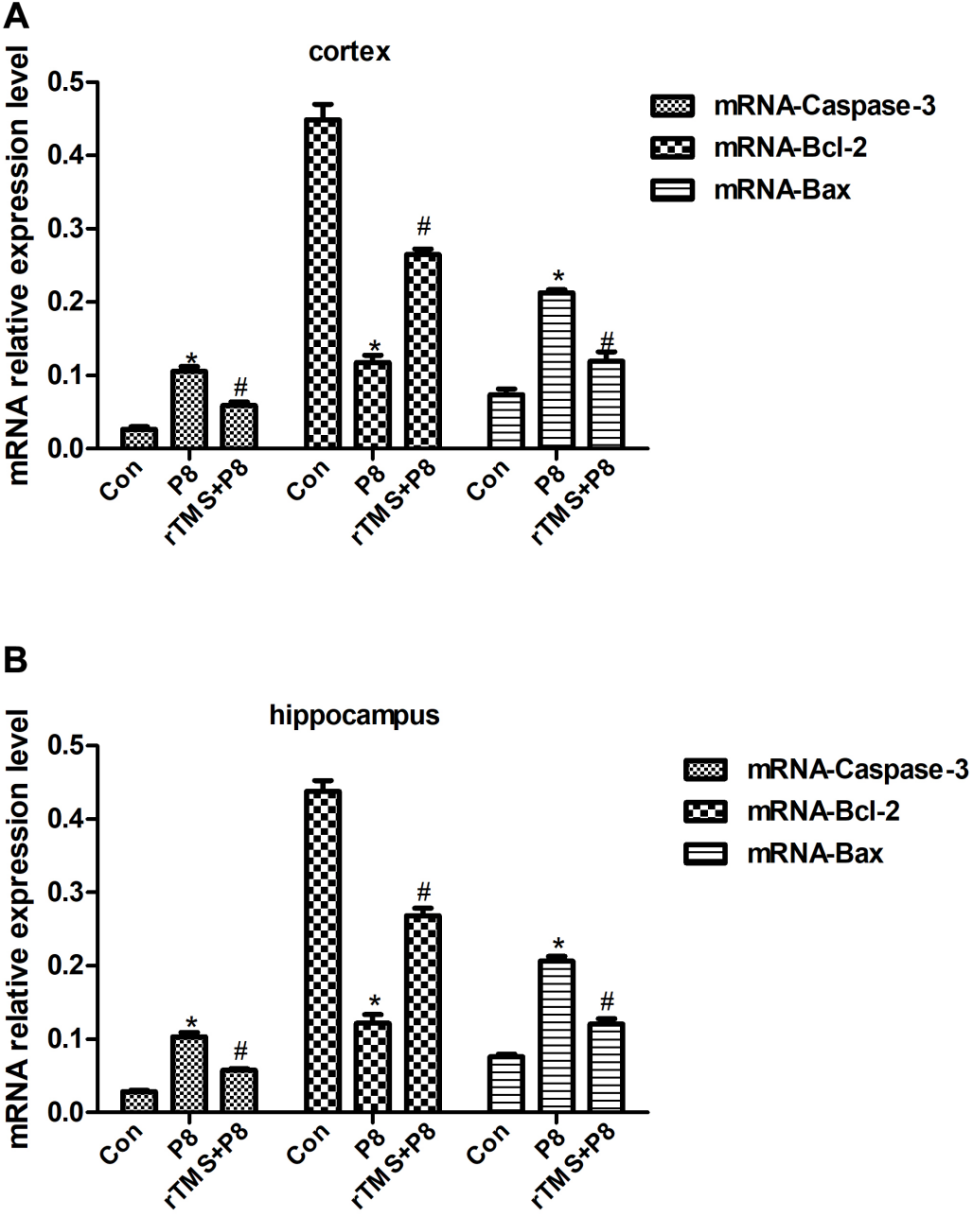
**Relative Caspase-3, Bcl-2 and Bax mRNA expression in cortical and hippocampal tissues of the in three groups of mice.** (**A**) Relative mRNA expression in mouse cortex. (**B**) Relative mRNA expression in mouse hippocampus. The data are presented as the mean ± standard deviation (n =5). *P < 0.05 vs. Con group; # P < 0.05 vs. P8 group.

Caspase-3, Bcl-2, and Bax expression levels were observed in 5 cortical and hippocampal tissue specimens by immunohistochemistry and qRT-PCR. Immunohistochemistry analysis of Caspase-3, Bcl-2, and Bax deposition demonstrated that in the cortex and hippocampus of P8 mice, the expression levels of Caspase-3 and Bax were significantly increased, while the expression of Bcl-2 was significantly decreased. After rTMS treatment, the expression of Caspase-3 and Bax decreased significantly, while the expression of Bcl-2 protein increased significantly (Figure 3). These findings indicated that P8 mice showed severe pathological features, significantly increased expression of apoptosis-related proteins (Caspase-3 and Bax) and decreased expression of an anti-apoptosis-related protein (Bcl-2) in the cortex and hippocampus, fortunately rTMS reversed this pathological change. Furthermore, qRT-PCR results showed that compared with the control group, Caspase-3 and Bax mRNA expression was significantly increased while the Bcl-2 mRNA expression level was decreased, and these trends were significantly reversed by rTMS treatment (Figure 4). The results indicated that the overexpression of proapoptotic proteins and the decrease in antiapoptotic proteins may be related to the cognitive function of P8 mice. rTMS could improve cognitive function by regulating the expression of these proteins and mRNAs.

### Involvement of the cAMP/PKA/CREB pathway in rTMS-mediated inhibition of neuronal apoptosis

**Figure 5:**
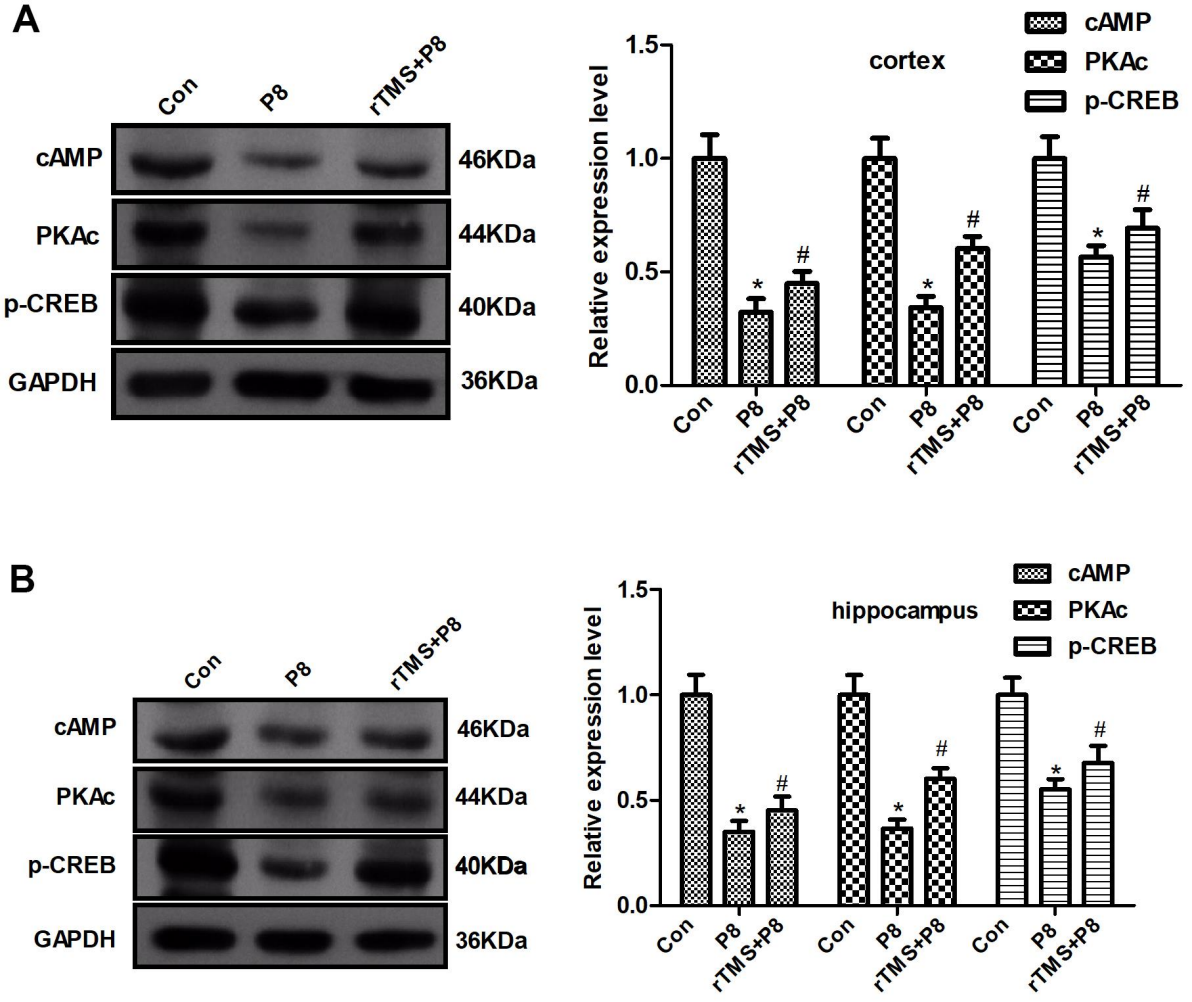
**Changes in the cAMP/PKA/CREB pathway in P8 mouse cortex and hippocampus after rTMS treatment.** (**A**) The expression of cAMP, PKAc and p- CREB in the mouse cortex was determined by western blot and quantitative analysis of cAMP, PKAc and p- CREB expression. (**B**) The expression of cAMP, PKAc and p- CREB in the mouse hippocampus was determined by western blot and the quantitative analysis of cAMP, PKAc and p- CREB expression. The data are presented as the mean ± standard deviation (n =5). *P < 0.05 vs. Con group; # P < 0.05 vs. P8 group.

To further determine whether the cAMP/PKA/CREB pathway participates in rTMS-related action, the expression of cAMP, PKAc, and CREB in mouse cortical and hippocampal tissue was determined by western blot. As shown in Figure 5, AD significantly decreased the cAMP, PKAc, and p-CREB expression, whereas rTMS increased the expression of cAMP, PKAc, and p-CREB. Taken together, these results demonstrated that the cAMP/PKA/CREB pathway might be involved in the rTMS-induced changes in cortical and hippocampal tissue.

## DISCUSSION

Several studies have shown that rTMS has a protective effect on the body, but the specific protective mechanism is not very clear [16]. Some studies have shown that rTMS may upregulate the expression of some protective mechanisms in the body, and simultaneously inhibit pro-cell injury mechanisms [17]. As an important second messenger in cells, cAMP plays an important role in the nervous system. Studies have confirmed that the cAMP/PKA/CREB signalling pathway is an important regulatory mechanism of the nervous system. The regulation of neuronal survival and axonal growth plays an important role, and may promote injured axon regeneration by inhibiting RhoA signalling pathways. Studies have shown that cAMP/PKA pathways are involved in mediating anti-inflammatory effects, and that inhibiting the cAMP/PKA pathways can aggravate mitochondrial metabolic disorders, leading to energy shortages and neuronal apoptosis [17–20].

rTMS is a magnetic stimulation method that has attracted the most attention in clinical medicine and neuroscience. Cotelli and Manenti et al. [21] used high-frequency rTMS to treat the dorsolateral prefrontal cortex in patients with AD and found that patients' naming ability was improved. In addition, high frequency rTMS has been found to have facilitate neurons that stimulate cortical nerve excitation [22]. The results of this study are consistent with this result, and the comparison between the P8 group and the control group after treatment was statistically significant, indicating that rTMS can significantly improve the learning and memory ability of AD. The effect of rTMS on the neural activity of learning and memory is a point at issue in brain science.

Studies have found that obvious apoptosis features in the hippocampus and cortex of AD mice, and the specific nerve tissues of patients with neurodegenerative diseases exhibit morphological changes of consistent with apoptosis [23, 24]. This evidence indicates that apoptosis is involved in the pathological process of AD. Thus, rTMS may inhibit neuronal cell apoptosis in the cortex and hippocampus of mouse brain tissue. Caspase is a specific apoptosis signal transduction molecule, and activated Caspase is the executor of apoptosis [25]. The activation of Caspases is manifested as a "waterfall" cascade reaction, and Caspase-3 is the most critical apoptotic executive protease that descends in the Caspase cascade reaction, and plays the final pivotal role in the apoptosis initiated by various programs [26]. This experiment indicated that the expression of Caspase-3 in the cerebral cortex and hippocampus of mice was significantly increased, which provided more substrates for Caspase-3 and activated the occurrence of the Caspase cascade. rTMS significantly reduce the expression of Caspase-3, and inhibited the initiation of apoptosis. In the apoptosis of nerve cells, the Bcl-2 family is indispensable [27]. Bcl-2 not only acts upstream of Caspase-3, but is also the direct substrate of Caspase-3, and both play a key role in apoptotic events [28]. Studies have shown that Bcl-2 can promote the cell cycle and cell proliferation, and Bax gene overexpression can accelerate cell apoptosis and counteract the inhibitory effect of Bcl-2 on cell apoptosis [29, 30]. The findings suggested that the expression of Bax in the cortex and hippocampus of AD model mice was significantly increased, while the expression of Bcl-2 was significantly reduced, but rTMS could significantly improve this change. The results of this experiment showed that apoptotic neurons increased, Bcl-2 decreased and Bax increased, in the cortex and hippocampus of AD model mice, indicating that Alzheimer's disease brain has a pro-apoptotic effect on tissues, and rTMS has an anti-apoptotic effect. This is in full agreement with the above Caspase-3 results.

As one of the important second messengers in the cell, cAMP plays an important role in the nervous system. Studies have shown that the cAMP/PKA/CREB signalling pathway is one of the important regulatory mechanisms of the nervous system, which regulates neuron survival and axon growth [19]. It may play an important role in inhibiting the RhoA signalling pathway to promote the regeneration of damaged axons. Inhibition of the cAMP/PKA pathway will aggravate mitochondrial metabolism disorders and lead to energy deficiency and neuronal apoptosis [20]. This study found that the Bcl-2 protein in the cerebral cortex and hippocampus of AD mice decreased, while the expression of Bax protein and Caspase3 protein increased. Morphological results showed that the cortex and hippocampal nerve cells were damaged. At the same time, cAMP and catalytic subunits were present in the cerebral cortex and hippocampus tissue. The expression of PKAc and p-CREB protein decreased, suggesting that the neurotoxicity of the cortex and hippocampus of AD mice may be related to the inhibition of the cAMP/PKA/CREB signalling pathway, further downregulate the anti-apoptotic gene Bcl-2 and upregulate the pro-apoptotic gene Bax, and then promote apoptosis. After rTMS treatment, morphological results and apoptosis-related proteins showed that the toxicity of nerve cells was reduced. At the same time, rTMS treatment also improved the inhibitory effect of AD mice in activating cAMP/PKA/CREB signalling pathway.

In our study, SAMP8 mice were used as the AD model. SAMP was selected by Japanese scholars for the breeding of natural AKP mutant mice and inbred and prolonged culture. SAMP consists of 9 sublines, among which SAMP8 is widely recognized as an ageing model of AD. SAMP8 has both the characteristics of natural ageing mice and learning and memory disorders similar to pathological changes of AD imagination. However, SAMP8 is considered unsuitable for long-term experiments because of its high cost and the short life span of the mice. In our study, apoptosis was not the primary reason of AD. However, in the present study, we aimed to investigate the effect of rTMS on cognition by inhibiting neuronal apoptosis. It is widely believed that the deposition of Aβ in the brain is the central link in the pathological changes of AD, which can trigger a series of pathological processes, that further promote the deposition of Aβ, thus forming a cascade reaction. Some studies have shown that Aβ has neurotoxicity, which can lead to nerve cell apoptosis, thus impairing learning and memory function [31, 32]. Therefore, by inhibiting neuronal apoptosis, memory decline may be delayed.

## CONCLUSIONS

The increase of in Bax levels, the decrease in Bcl-2 levels and the activation of downstream Caspase-3 in AD mice induced the apoptosis of cortical and hippocampal nerve cells. rTMS could improve the changes of Bax and Bcl-2 and the activation of downstream Caspase-3, promote neuroprotection, and activate the cAMP/PKA/CREB signalling pathway. One of the mechanisms by which rTMS reverses the neurotoxicity of Alzheimer’s disease in mice may be by reversing the downregulation of the cAMP/PKA/CREB signalling pathway, which may be related to the mitochondrial energy metabolism of nerve cells. This phenomenon is worthy of further research.

## MATERIALS AND METHODS

### Experimental animal and modeling

Male 6-month-old 50 SAMP8 and 20 SAMR1 mice (30±2g) were provided by Experimental Animal Center of North China University of Science and Technology and adaptively housed in the animal center for one week. The mice were housed separately under an alternative 12 h day/night cycle. The temperature and humidity in the were cab kept in a certain range (23 ± 2° C and 50–65%, respectively). Before the experiment, all mice were fasted for 12 to 16 hours, with free water intake. In fact, 10 mice were excluded from this study, and 60 qualified mice were used in the following study (excluding those with visual impairment, motor impairment and body mass). The study was approved by the Ethics Committee of North China University of Science and Technology.

### Grouping

A total of 60 mice were randomly divided into three groups: the control group (Con, n =20), SAMP8 group (P8, n=20), and the rTMS+SAMP8 group (rTMS+P8, n=20). The rTMS+P8 group received 10 groups of rTMS with a frequency of 5 Hz on the top of the skull every day, with an interval of 20 s between each group, a total of 1000 stimulation pulses, and a stimulation intensity of 1.2 T, for 14 consecutive days. All studies followed the blind principle and after behavioral and histological analysis was completed, the animal codes were revealed.

### Morris water maze test

The pool was a round stainless steel sink with a diameter of 90 cm (with a black platform submerged 2 cm under the surface, diameter: 10cm; height: 28cm), and the tank was filled with water (22-25° C) to a depth of 30 cm. The test was conducted for 5 days after 14 consecutive days treatment. The tank was randomly divided into 4 quadrants of EN, EW, SW and SN. The mice were placed into the selected pool position, and the swimming time and trajectory of the mice were recorded. The mice were given 60s to find and climb on the platform stage. If the animal did not find the platform within 60 s, the experimenter led the animals to the platform. The time it takes for the animals to find the platform is called the “escape latency”. The first day was for training, with one training in the morning and one in the afternoon. The test was conducted 4 days after the end of treatment, and each mouse was tested four times a day for four consecutive days. The swimming speed was evaluated on the last day of the test, when the platform was removed. The average escape delay for a total of four trials was calculated.

### Y maze test

The training was carried out in a three-part radial Y maze. The top of each arm was equipped with a 15-watt light bulb, and the bottom of the box was a parallel copper rod. The bottoms of the two dark arm boxes were energized, and lights illuminate the safe area. The mice were placed into the Y maze to adapt for 5 min before applying electrical stimulation (the stimulation parameters were set as: voltage 60 V, and delay 5 s). During training, the safe zone is randomly changed, with the correct response of the mouse to escape to the safe zone was after receiving an electric shock stipulated, and the mice were allowed to stay in the safe zone for 30 s to consolidate memory. When the rat had 9 correct responses (9/10) in 10 consecutive training sessions, this indicated a learning grid. The number of training required before the mouse reaches the 9/10 standard is recorded to quantify the performance of the mouse when it was qualified. Memory ability test: 24 h after the training, the mice were trained 10 times according to the above method, and the number of correct responses was recorded as the memory score to represent the level of memory ability.

### Histopathological examinations

After the MWM test, cortical and hippocampal tissues of mice in all three groups were removed immediately at 24 h after reperfusion. Then the mouse brain specimens fixed with 10% formaldehyde were conventionally dehydrated, soaked in wax, embedded and sectioned. The sections were stained with hematoxylin and eosin (HE) for histopathological examination to observe the morphological changes of neurons in an optical microscope under 40× magnification(10x eye piece x 40x objective) in the hippocampus of mice.

### Immunohistochemistry

Cortex and hippocampal tissues were exposed to 4% paraformaldehyde at 4° C and 30% sucrose for 2 hours respectively, embedded in paraffin and sectioned at 5μm immunohistochemistry experiments. The slices were washed three times with 0.01mol/L TBS buffer, and incubated at room temperature for 1 h in blocking buffer. After that, the slices, at 4° C, were incubated with primary antibodies including Caspase-3 (Abcam, USA, ab184787,1:1000), Bax (Abcam, USA, ab32503,1:1000), and Bcl-2 (Abcam, USA, ab182858, 1:1000) overnight. Slices were washed three times for 10 min each in PBS at room temperature, and then incubated with HRP-labelled goat anti-mouse secondary antibody at 37° C for 30min. DAB chromogenic agent was used for colour development. Slices were rinsed thoroughly with tap water, re-stained, dehydrated, made transparent as necessary, and sealed. The positive cells were observed under a microscope. We used Image ProPlus software to do statistical analysis.

### TUNEL staining

TUNEL (terminal deoxynucleotidyl transferase (TdT)-mediated dUTP nick end labelling) staining was used to investigate the role of rTMS in neurons of cortex and hippocampus induced by AD. The mouse cortex and hippocampus specimens fixed with 10% formaldehyde were conventionally dehydrated, soaked in wax, embedded and sectioned, and cut into slices. The sections were deparaffinized in xylene, hydrated, digested with trypsin, and stained in TUNEL mix. Nest, the slices were dehydrated, made transparent, sealed with neutral gum. Finally, the sections were observed with an optical microscope and the apoptosis rate of cortical and hippocampal neurons was calculated.

### Quantitative real-time PCR analysis

The expression levels of the mRNAs (Caspase-3, Bax, and Bcl-2) were quantified using qRT-PCR analysis. According to the manufacturer’s instructions, the total RNA of the mouse cortex and hippocampus was isolated with TRIzol reagent, and then cDNA was obtained by reverse transcription. qRT-PCR was performed as previously described [33]. Calculations were measured using the 2 −ΔΔCt method to analyze the relative expression of each gene, and the bar chart was drawn using GraphPad Prism 5.0. The primers were as follows: Caspase-3: forward: 5’-CCGCTTATAACTGTTGCTGT-3’, reverse: 5’-TTCCCAGCGGTCCGCTTCAT-3’; Bax: forward: 5’-CCCAGAGGCGGGGTTTCA-3’, reverse: 5’-GGAAAAAGACCTCTCGGGGG-3’; Bcl-2: forward:5’-CATATCTGTTTCGAGAATCA-3’, reverse: 5’-CACCCGTTTCTCCGATAAGCA-3’; β-actin: forward: 5’-CTTCGCTTTCGAACAT-3’, reverse: 5’-CCACATATTCCTCCAACTGACC-3’.

### Western blot

The mouse cortex and hippocampal tissue were rapidly isolated on ice, total proteins were extracted, the protein concentration was determined by the BCA reagent total proteins were extracted and the concentration was determined by BCA reagent (Abcam, USA, ab146331). The samples were subjected to SDS-PAGE electrophoresis, and the separated proteins were transferred to PVDF membranes. The blots were blocked with 5% skim milk at room temperature for 1 h, and then the membrane was incubated with the primary antibody overnight at 4° C, including anti-cAMP (Abcam, USA, ab76238, 1:2000), anti-PKA (catalytic subunits) (Abcam, USA, ab59218, 1:1000), anti-CREB (Abcam, USA, ab32515, 1:500). Then, the cells were incubated with horseradish peroxidase conjugated goat anti-rabbit IgG H&L (Abcam, USA, ab6721,1:5000) for 2 h at room temperature. After incubation with a properly titrated secondary antibody, the cells underwent ECL luminescence development. The developed image was scanned to a computer for analysis after exposure.

### Statistical analysis

The data in the experiment are expressed as the mean±standard deviation (S.D.) and analysed by SPSS 20.0. There was a minimum of five mice per group. One-way ANOVA was used to analyze the results followed by the Newman-Keuls post-hoc test. A P value of < 0.05 was considered significant for all statistical analyses.

### Ethics statement

The mouse experiments were approved by the Animal Ethics Welfare Committee of North China University of Science and Technology.
